# Green Extraction of Carrageenans from *Mastocarpus stellatus*

**DOI:** 10.3390/polym14030554

**Published:** 2022-01-29

**Authors:** Noelia Flórez-Fernández, Elena Falqué, Herminia Domínguez, María Dolores Torres

**Affiliations:** 1 CINBIO, EQ-2 Group, Facultade de Ciencias, Campus Ourense, Universidade de Vigo, As Lagoas, 32004 Ourense, Spain; noelia.florez@uvigo.es (N.F.-F.); herminia@uvigo.es (H.D.); 2 Departamento de Ingeniería Química, Facultade de Ciencias, Campus Ourense, Universidade de Vigo, As Lagoas, 32004 Ourense, Spain; 3 Departamento de Química Analítica, Facultade de Ciencias, Campus Ourense, Universidade de Vigo, As Lagoas, 32004 Ourense, Spain; efalque@uvigo.es

**Keywords:** subcritical water extraction, hot water extraction, carrageenan, antioxidant, mechanical properties, antitumoral

## Abstract

The recovery of biopolymers from natural resources using eco-friendly extraction technologies that enhance their mechanical properties has gained attention in recent years. In this context, this work deals with the isolation of hybrid carrageenans from *Mastocarpus stellatus* red seaweed using subcritical water extraction operating in a wide range of thermal conditions (70–190 °C). The extracted biopolymers were analyzed by means of either Fourier-Transform infrared, nuclear magnetic resonance, rheological or cell viability assays. In parallel, the fundamental chemical composition of the seaweed used as raw material, as well as the main phytochemical properties of the soluble liquid extracts, were also studied. Results indicated that thermal extraction conditions significantly affected the rheological behavior of the recovered hybrid carrageenans. The hybrid carrageenan extraction yields varied, with results between 10.2 and 30.2% being the highest values obtained at hydrothermal treatment of 130 °C. A wide palette of viscous features was identified for recovered hybrid carrageenans, with the strongest rheology properties observed at the same temperature. It should be remarked that the maximum inhibitory effect was also obtained at 130 °C for both the ovarian carcinoma cell line (A2780) (65%, IC_50_: 0.31 mg/mL) and lung carcinoma cell line (A549) (59%, IC_50_: 0.41 mg/mL).

## 1. Introduction

Polymers from macroalgae have a great potential in different fields, including pharmaceutical, food or cosmeceutical fields [1,2,3,4,5]. The classification is based on the pigmentation, but also other compounds such as polysaccharides are found in the cell wall of seaweed. In this context, the main polymer of green seaweeds (Chlorophyta) is ulvan, the main polymer of red seaweeds (Rhodophyta) is carrageenan and and the main polymer of brown seaweeds (Phaeophyceae) is fucoidan [6]. Specifically, *Mastocarpus stellatus* is a red seaweed and its main polymer, carrageenan, is essentially comprised of D-galactopyranose units. Six basic forms have been defined *iota*-, *kappa*-, *lambda*-, *mu*-, *nu*- and *theta*-carrageenan but the most common are *iota*-, *kappa*- and *lambda*-carrageenan [7]. Sulfate ester groups were also found in their structure, one for *kappa*-, two for *iota*- and three for *lambda*-carrageenans. The viscous and elastic properties of these polymers have a rheological behavior, which is potentially of interest for different industries; *kappa*- and *iota*- have gelling properties while *lambda*-carrageenan has thickening properties [7]. However, different parameters associated with the origin, harvesting season, sea temperature and other factors can influence the composition and, as a result, the features and behavior of the carrageenans [8].

Several extraction methodologies have been developed to extract polymers, which is potentially of interest, from the cell wall of the seaweeds [9]. Nowadays, it is possible to use eco-friendly technologies that focus on the use of water as an extraction agent; these strategies are an important factor in recovering biopolymers from marine biomass. In this context, pressurized hot water is an extraction technology previously used with different raw materials, such as vegetables [10], different seaweeds [11] or even flowers [12], among others.

Bioactive compounds have also been found in polymers from red seaweeds. Some of the properties are antioxidant activity [13], prebiotic activity [14] and others. In this context, biomedical applications such as anti-inflammatory activity [15], antiviral activity [16], antibacterial activity [17], antimicrobial activity [18] and antitumoral activity [14], among others, [1] have been reported; particulate systems with biomedical applications could also be another possibility for delivering compounds that are of in this field [19,20].

The parameters associated with rheological study are a key factor in finding the suitable mechanical properties that focus on the possible applications of these polymers, not only during the manufacturing and processing but also in the storing step [21,22]. Several studies on the mechanical features of hybrid carrageenans from different sources and extraction treatments have been published in the past decade [23], which demonstrates the relevance of this aspect.

The aim of this work was to produce high valuable compounds, biopolymers as carrageenans with different rheological properties and bioactive compounds such as antioxidants and phenolic compounds using an ecofriendly extraction technology known as pressurized hot water extraction, in the absence of alkali solvents during the extraction process that can modify their rheological profiles.

## 2. Materials and Methods

### 2.1. Raw Material

*M. stellatus* red seaweed was supplied by the enterprise Porto-Muiños (A Coruña, Spain). The fresh seaweed was collected in March 2018 and sent to the laboratory where the batch used for this work was cleaned and washed with tap water. The moisture content was determined gravimetrically, with the knowledge that their content was 74.4% d.b. (dry basis). In order to be used for the experimental process, the seaweed was ground (0.5 cm aprox.) and stored in hermetic plastic bags in the dark at −18 °C.

### 2.2. Extraction Procedures

#### 2.2.1. Pressurized Hot Water Extraction Process

The red seaweed (before cleaned and grinded) was mixed with distilled water using a 30:1 liquid:solid ratio (*w*/*w*) in accordance with previous work [24]. This mixture was introduced in a stainless-steel pressurized reactor (Parr Instruments series 4842, Moline, IL, USA) equipped with a stirred vessel. In brief, the *M. stellatus* red seaweed and water formed a suspension, the range of temperatures to explore was 70 to 190 °C. The reactor was heated until it reached the selected temperature, and when the temperature was reached the reactor was quickly cooled until it returned to room temperature. Two phases were obtained by vacuum filtration: a liquid and a solid phase. The liquid phases recovered at the different temperatures tested were chemically studied. Furthermore, these liquid phases were also used for the recovery of carrageenan.

#### 2.2.2. Isolation of Carrageenan

Carrageenan was obtained by precipitation from the liquid phase after the hydrothermal extraction process of adding ethanol (96%), using the ratio 1:1.5 for liquid sample:ethanol (*v*/*v*). No alkali treatment was used during extraction process in order to not modify the natural rheological properties of the extracted biopolymers. The precipitates were separated by filtration under vacuum, and the solid residues were washed with ethanol (twice); the samples were dried at 40 °C for around 24 h in an oven. Samples obtained were milled and stored at room temperature in the dark in hermetic plastic bags until further analysis.

In order to facilitate the monitoring of the process developed in this work, a flow diagram in the results section exhibits an overview of the extraction treatments used, the phases obtained and the corresponding characterization.

### 2.3. Characterization of the Raw Material

#### 2.3.1. Proximal Characterization of the Seaweed

The macroalga *M. stellatus* was chemically characterized. Moisture content was determined at 105 °C for 48 h in a laboratory oven, and ash content was determined after calcination at 575 °C for 6 h in a muffle, both moisture and ash content, were gravimetrically analyzed. Total nitrogen was determined by the Kjeldahl method, and the results were converted to protein using the factor 4.59 ± 0.54, which is specific for red seaweed [25].

#### 2.3.2. Metals Determination

To determine the content of Zn, Ca, Mg, Fe, Cu, As, P, Cr, Cd, Pb, I and Hg, a digestion of the samples was performed in a Marsxpress microwave (CEM, Tacoma, WA, USA); the ashes of the sample were digested with HNO_3_ and H_2_O_2_ at 1600 W for 15 min and 200 °C for 10 min. Different technologies were used to analyze the samples: Na and K were determined by the Atomic Emission Spectrophotometry (AES). The Atomic Absorption Spectrophotometry (AAS) was used to determine Zn, Ca, Mg, Fe, Cu, As and P using a 220 Fast Sequential Spectrophotometer (Varian, Palo Alto, CA, USA). The Inductively Coupled Plasma Mass Spectrometry (ICP-MS) (X Series, Thermo Scientific, Waltham, MA, USA) was used to determine Cr, Cd, Pb and I and, finally, Hg was analyzed by the Cold Vapor-Atomic Absorption Spectroscopy (CVAAS).

To determine the content of Zn, Ca, Mg, Fe, Cu, As, P, Cr, Cd, Pb, I and Hg, a digestion of the samples was performed in a Marsxpress microwave (CEM, Tacoma, WA, USA); the ashes of the sample were digested with HNO_3_ and H_2_O_2_ at 1600 W for 15 min and 200 °C for 10 min. Different technologies were used to analyze the samples: Na and K were determined by the Atomic Emission Spectrophotometry (AES). The Atomic Absorption Spectrophotometry (AAS) was used to determine Zn, Ca, Mg, Fe, Cu, As and P using a 220 Fast Sequential Spectrophotometer (Varian, Palo Alto, CA, USA). The Inductively Coupled Plasma Mass Spectrometry (ICP-MS) (X Series, Thermo Scientific, Waltham, MA, USA) was used to determine Cr, Cd, Pb and I and, finally, Hg was analyzed by the Cold Vapor-Atomic Absorption Spectroscopy (CVAAS).

#### 2.3.3. Sulfate Determination

Sulfate content was analyzed using a method based on ionic chromatography (Metrohm Advanced IC-861, Herisau, Switzerland) fitted with a Metrosep A Supp 5–250 column (250 × 4 mm). It was during a mobile phase with 3.2 mM of sodium carbonate/1 mM of sodium bicarbonate (0.70 mL/min) equipped with an IC-819 detector [26]. All above measurements were performed in at least triplicate.

### 2.4. Physicochemical Characterization of the Hydrothermal Carrageenan-Free Liquid Phases

Several physicohemical features (pH, soluble sulfate content, soluble protein content, phenolic content, antiradical assays, oligossacharides, profile of molar mass distribution) were measured in the liquid phases obtained after carrageenan extraction, as describe below.

#### 2.4.1. pH

Liquid phases obtained by water extraction from red seaweed *M. stellatus* were studied, pH determination, previously calibrated, was carried out using a GLP 21 pH meter (Crison, Barcelona, Spain).

#### 2.4.2. Soluble Sulfate Content

The gelatin-barium chloride method [27] was used to analyze the soluble sulfate content. Briefly, samples were hydrolyzed with trichloroacetic acid (4%) (Sigma-Aldrich, San Luis, MO, USA). Afterwards, gelatin-BaCl_2_, previously prepared, was added and mixed using a vortex. After being incubated for 15 min at room temperature, the absorbance was measured at 500 nm.

#### 2.4.3. Soluble Protein Content

Soluble protein was quantified by the Bradford method [28]. The samples were mixed with the Bradford reagent (Panreac, Madrid, Spain) and incubated for 5 min at room temperature. The absorbance was measured at 595 nm. The standard curve was carried out with bovine serum albumin (BSA) (Sigma-Aldrich, San Luis, MO, USA).

#### 2.4.4. Phenolic Content

The total phenolic content was analyzed following the reported protocol [29]. Briefly, samples were mixed with Folin-Ciocalteu reagent (1 N) and sodium carbonate at 20%, an incubation period in the dark at room temperature for 45 min was necessary. The absorbance was measured at 730 nm, distilled water was used as blank, and the standard curve was carried out with gallic acid (Sigma-Aldrich, San Luis, MO, USA).

#### 2.4.5. Antiradical Properties-TEAC Value

The antioxidant capacity was determined following the protocol previously reported [30]. The ABTS radical cation (ABTS^+^) [2,2-azinobis(3-ethyl-benzothiazoline-6-sulfonate)] was expressed as a TEAC value (Trolox Equivalent Antioxidant Capacity). The absorbance was measured at 734 nm after being incubated (samples mixed with diluted ABTS^+^ solution) for 6 min. The standard curve was realized using Trolox as a pattern (6-hydroxy-2,5,7,8-tetramethylchroman-2-carboxylic acid, Sigma-Aldrich, San Luis, MO, USA).

#### 2.4.6. Oligosaccharides Composition

Liquid phases free of carrageenan obtained from the red seaweed *M. stellatus* using hot water extraction were analyzed to determine the content of oligosaccharides. At first, a dialysis step to remove the salts of the samples was necessary (molecular weight cut-off (MWCO): 100–500 Da, SpectrumLabs, San Francisco, CA, USA). In order to quantify the saccharide fraction, a post-hydrolysis step was necessary, and samples were processed at 121 °C for 30 min in an acidic medium (H_2_SO_4_, 4%). Afterwards, the samples were filtered through membranes (0.45 μm) and analyzed using high performance liquid chromatography (HPLC) 1100 series (Agilent, Germany). The column used was an Aminex HPX-87H (300 mm × 7.8 mm, BioRad, Hercules, CA, USA) operating at 60 °C during the mobile phase, H_2_SO_4_ (0.003 M) was added at a flow rate of 0.6 mL/min and the patterns were glucose, galactose and fucose (Sigma-Aldrich, San Luis, MO, USA).

#### 2.4.7. Profile of Molar Mass Distribution

The dialyzed liquid phases after water extraction process (in the absence of carrageenans) obtained at different temperatures were evaluated to determine the profile of molar mass distribution by High Performance Size Exclusion Chromatography (HPSEC). A High-Performance Liquid Chromatograph (HPLC) from Agilent (Germany) was required to analyze this feature. The equipment was provided by two columns from Tosoh Bioscience (Germany), both were placed in series (300 mm × 7.8 mm TSKGel G3000PW_XL_ and 300 mm × 7.8 mm TSKGel G2500PW_XL_) and a guard-column was also positioned in front (PWX-guard column, 40 mm × 6 mm) fitted by a refractive index (RI) detector. Columns were working at 70 °C and the flow of the mobile phase (Milli-Q water) was 0.4 mL/min. In order to establish a pattern, dextrans (DX) at a molecular weight of between 1000 and 80,000 g/mol from Fluka (USA) were used.

### 2.5. Carrageenans Characterization

#### 2.5.1. Fourier-Transform Infrared (FT-IR) Spectra

Carrageenans extracted from the liquid phases obtained at 80, 100 and 170 °C were lyophilized (Christ Alpha 2–4 LD plus, Frankfurt, Germany), in accordance with the tested systems [31]. The samples were mixed with potassium bromide to prepare a that is table suitable for analysis by FT-IR (Nicolet 6700, Thermo Scientific, Waltham, MA, USA); the detector used was DTGS KBr and the source refraction index. The software used to acquire the results was OMNIC. The spectra range for the samples was 500 to 2000 cm^−1^. The spectral resolution of the equipment was: 4 cm^−1^ and 32 scans/min. All the samples were assessed at least in duplicate.

#### 2.5.2. Proton Nuclear Marnetic Resonance (NMR)

^1^H-NMR of the extracted carrageenans was performed with an ARX400 spectrometer (Bruker BioSpin GmbH, Bremen, Germany). Biopolymer solutions were carried out at 10 mg/mL employing deuterated water as solvent. The internal standard used was 3-(trimethylsilyl)-L-propane sulfonic acid (Sigma-Aldrich, San Luis, MO, USA). The operation conditions were 75 °C and 400 MHz. The signals of the samples were identified, and the corresponding kappa/iota ratio was calculated.

#### 2.5.3. Rheological Testing

Rheological testing focused on the impact of the proposed hydrothermal treatment on the viscous behavior of recovered carrageenans, analyzing the correspondence between experimental data from steady shear and oscillatory measurements. Carrageenans extracted after hydrothermal treatment at 70–190 °C were dispersed in hot water (80 °C for 1 h) at a commonly used biopolymer content (1.0, 2.0%) and fixed ionic strength 0.1 M (potassium chloride). The biopolymer and salt content were selected following the indications previously reported for these hybrid carrageenans according to the corresponding sol/gel transition diagrams [21,32]. It should be indicated that vigorous stirring (around 3500 rpm) was required to ensure the fill biopolymer dissolution. Then, samples were cold stored for 24 h to ensure complete maturation of the developed gels [32]. The apparent and complex viscosity of the above systems was determined using a controlled-stress rheometer (MCR 302, Paar Physica, Graz, Austria), which uses sand blasted plate-plate geometry (1 mm gap, 25 mm diameter). Samples were placed on the measuring system at 25 °C, the edges were sealed with light paraffin oil and were rested for 5 min. Apparent viscosity profiles (viscosity, η, vs. shear rate, γ) at 25 °C were obtained using steady shear measurements by decreasing the shear rate following a logarithmic ramp. At the same temperature, complex viscosity profiles (viscosity, η*, vs. angular frequency, ω) were determined using oscillatory measurements in terms of frequency sweeps (25 °C, 10 Pa) within the linear viscoelastic regime (<45 Pa for above carrageenan systems).

### 2.6. Cell Viability of the Free-Carrageenan Liquid Phases and the Carrageenans

The culture medium RPMI (Roswell Park Memorial Institute) 1640 and DMEM (Dulbeco Modified Eagle’s Medium-low glucose) were supplemented with FBS (Fetal Bovine Serum) at 10% and L-glutamin at 2 mM, these mediums were used for cultured ovarian carcinoma cells (A2780) and lung carcinoma cells (A549), respectively, in an atmosphere of air/CO_2_ (95%/5%) at the temperature of 37 °C. The 3-[4,5-dimethylthiazol-2-yl]-2,5-diphenyltretrazolium bromide (MTT) test was performed to study the cell growth inhibition, which is worked out according to its change to formazan by viable cells. The cells A2780 and A549 were seeded in a 96-well plate with a density of 4000 cells/well and 5000 cells/well, respectively. The time of incubation was 24 h for A2780, whereas for A549, it was 72 h. After that, 5 mg/mL (10 µL) of MTT dissolved in PBS was added to each well. The cell plates were again incubated for 4 h, after this time, SDS at 10% (100 µL) solution in 0.01 M HCl was added. The cell plates were incubated for 12–14 h. The absorbance of the cell plates was read at 595 nm (Tecan Infinite M1000 Pro, Viena, Austria).

The growth inhibition percentage and the inhibitory potency were calculated according to Equations (1) and (2), respectively:

(1) $$Growth\;inhibition\;\left(\%\right)=100-\left(\frac{AO}{AT}100\right)$$

(2) $$Inhibitory\;potency\;\left(\%\right)=\frac{E_{max}}{1+{\left(\frac{IC_{50}}{EConc}\right)}^{n}}\;100$$

where, in Equation (1), *AO* is the absorbance of the extract and *AT* the absorbance of the water. In the case of the *inhibitory potency*, it is calculated vs the extract concentration represented in the equation by *EConc*, the maximum inhibitory effect in the equation is *Emax*, the concentration inhibiting growth by 50% is represented by *IC*_50_ and *n* is the slope.

### 2.7. Statistical Analysis

All of the above experimental measurements were made in at least triplicate. One-factor analysis of variance was employed to statistically analyze the data using PASW Statistics v.22 (IBM SPSS Statistics, New York, NY, USA) software. Whenever means differences were identified by variance analysis, a *post-hoc* Scheffé test was performed to discriminate means (95% confidence, *p* < 0.05). In the study of cell viability, non-linear regression was carried out with GraphPad Prism Version 2.01, 1996 (GraphPad Software Inc., San Diego, CA, USA).

## 3. Results and Discussion

**Figure 1:**
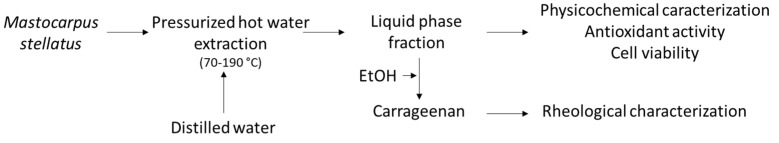
The extraction process using pressurized hot water extraction of the edible red seaweed, *M. stellatus*.

Figure 1 presents a flow diagram of the extraction process for *M. stellatus* red seaweed using the extraction technology known as compressed hot water or pressurized hot water. In this process, only distilled water was used as a solvent to obtain compounds of commercial interest using an eco-friendly extraction technology.

### 3.1. Raw Material

**Figure 2:**
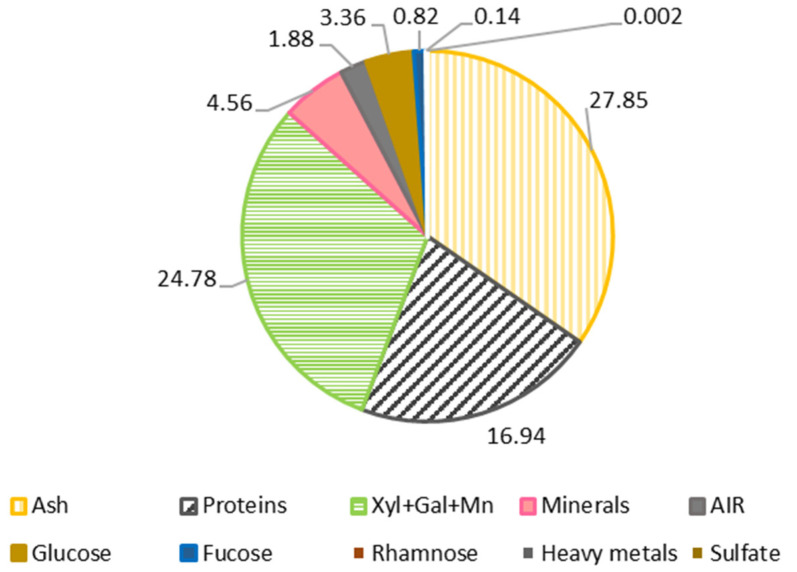
Proximal composition of the edible red seaweed *M. stellatus*. Standard deviations were lower than 4.5% in all cases.

The composition of the raw material, *M. stellatus*, was exhibited in Figure 2. The maximum percentage was attributed to ashes (27.9%), followed by xylose, galactose and mannose (24.8%), sulfate (19.7%), protein (16.9%), minerals (4.6%), glucose (3.4%), acid insoluble residue (AIR) (1.9%), and fucose, rhamnose and heavy metals (1%). Gómez-Ordóñez et al. [26] also studied the composition of the red seaweed *M. stellatus*, the results obtained were in harmony with this work; the values for ash content were 24.99%, protein 21.3% and the total sugar content was 19%. In other work, the ash of the seaweed *M. stellatus* made up 25% and sulfate content mad up 57.1%, which is more than double [33]. These differences could be due to the season in which the seaweed was collected and other environmental factors.

### 3.2. Physicochemical Characterization of Carrageenan-Free Liquid Phases after Hydrothermal Extraction Process

#### 3.2.1. Soluble Sulfate Content

Table 1 contain the results of the soluble sulfate content in the liquid phase obtained after the extraction process at different temperatures varying from 70 to 190 °C. The results show an increase until the temperature reached 90 °C, then a decrease and at 170 °C another increase; this behavior could suggest a split interpretation according to the temperatures, between 70 °C and the boiling point of water (100 °C), where an increase and decrease in the results is observed. A similar conduct is present in the data between 130 °C and 190 °C, an increase follows a decrease. 

According to the literature, a higher content of sulfate was found in comparison with brown seaweeds, which could be due to the sulfate present in the structure of the carrageenans [34]. A sulfate content of around 45% was measured in the liquid phase obtained at 90 °C, which decreased at 100 °C to become 39%. However, at 170 °C, a quantity of almost 48% was measured; a sharp decreasing was observed at 190 °C where the sulfate content was 11%. This behavior could be due to the temperature of extraction. It could also be due to the fact that when the temperature increases, the pH decreases from 7.35 to 6.17. This slight difference is due to the hydrogen ions presents in the liquid phase, it is probable that the high temperature of extraction causes a loss of hydrogen and sulfate groups that are associated with the polymeric chain. In other work, the authors obtained a 30% sulfate content at the maximum irradiation power tested (500 W) using the same seaweed but different extraction technology, namely microwave hydrodiffusion and gravity (MHG) [35].

#### 3.2.2. Soluble Protein Content

The values of protein content presented a slight variation between 0.71 and 1.27 (Table 1). Two different behaviors are observed; an increase up to 100 °C results in the maximum value (1.27%). After this temperature was reached, the content decreased to 0.79% (130 °C), then increased to 0.84% at 190 °C; the maximum temperature tested. Other work, where *M. stellatus* was also used as raw material, exhibit similar values. In this case, using MHG extraction technology [35].

#### 3.2.3. Phenolic Content

Table 1 shows the total phenolic content (expressed as gallic acid) present in the extracts. Between 70 and 90 °C, the quantity of gallic acid was stable at around 0.25%; however, when the temperature was increasing, the content was decreasing. The temperature could be associated with the degradation of these compounds. Other work, using the same raw material but different extraction technology based on the irradiation of the sample, showed an increase in the phenolic content when the irradiation was increasing, where the maximum value was beyond the 20 mg/g extract [35].

#### 3.2.4. Antiradical Properties-TEAC Value

The TEAC value was used to determine the antiradical properties of the samples obtained; the results presented similar behavior to that of the gallic acid content (Table 1). The maximum values were obtained between 70–90 °C (3.2 and 3.8%, respectively), whereas at higher temperatures, a decreasing of the antioxidant activity was observed. An exception was at 190 °C where a similar value to that at 90 °C was observed. Other authors have performed two different extractions; one was at 3 °C overnight and the other was at 45 °C for 45 min. The values obtained were in harmony and were 4.5% [36].

#### 3.2.5. Oligosaccharides Composition

Table 1 also included the oligosaccharides content present in the samples of the *M. stellatus* red seaweed. The values increased from 27% to 62% according to the increase in the temperature; at 190 °C the result reduced to 44% (from 62% at 170 °C). This behavior could be explained by the fact that at high temperatures, a degradation of the saccharide fraction could take place. In other work, where high temperatures (120 to 240 °C) were used to extract compounds from seaweed, the same behavior was exhibited [37,38]. Gómez-Ordóñez et al. [39] studied the carbohydrate content after extractions under different conditions: with the water at 22 °C and 60 °C, with an acidic medium using HCl (0.1 M), and adding a basic medium KOH (2 M); this allowed them to obtain several fractions. These fractions were characterized, and the values are in harmony with the results obtained at a low temperature (70 °C); this could be because the cell wall, at that temperature, does not allow the release of these compounds, meaning that it is necessary to apply more severe conditions.

#### 3.2.6. Profile of Molar Mass Distribution

**Figure 3:**
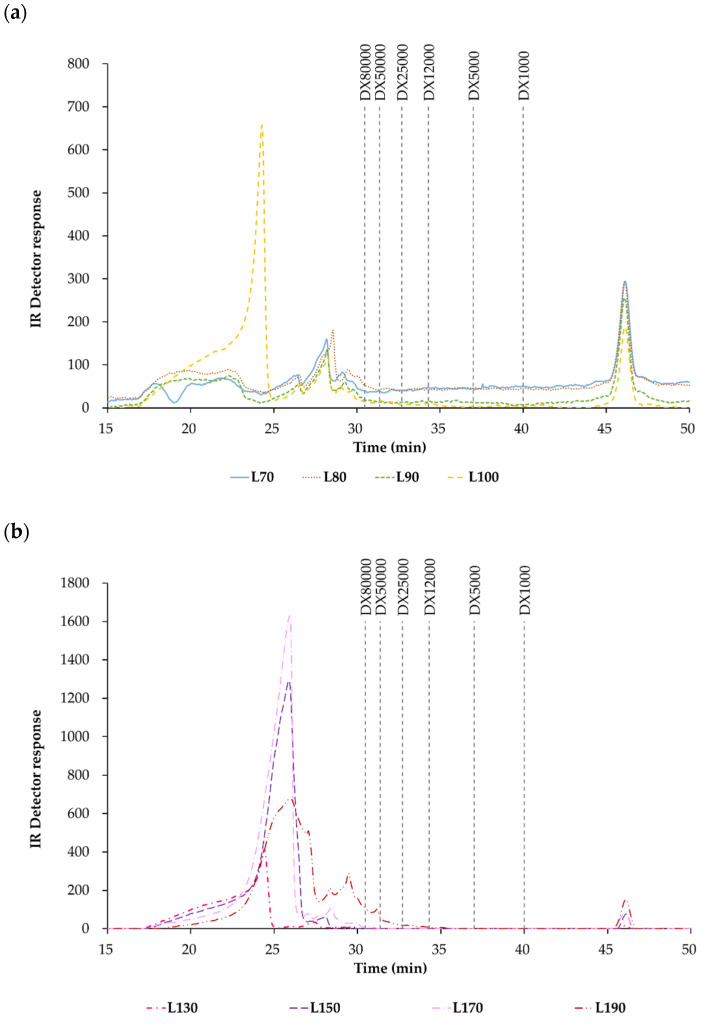
Molar mass distribution profiles determined by HPSEC from carrageenan-free liquid phases obtained by pressurized hot water extraction (L) at the temperatures 70, 80, 90 and 100 °C (**a**) and at 130, 150, 170 and 190 °C (**b**). Note: DX means dextran from 1000 g/mol to 80,000 g/mol.

Figure 3 exhibits the molar mass distribution profiles from the carrageenan-free liquid phases obtained using pressurized hot water extraction. Figure 3a makes reference to autohydrolysis carrageenan-free liquors from 70 to 100 °C, in both cases, a peak above 80 kDa is observed and, in the first case, a peak below 1 kDa is also shown. The applied temperature could be responsible for this behavior; at 100 °C a change in the pattern was observed, which is more similar to the patterns that occur between 130 and 190 °C (Figure 3b), where a significant change could be expected; therefore, (a) and (b) are in harmony. Temperatures between 130 and 190 °C presented a very small peak below 1 kDa; the important changes in the profiles obtained above 80 kDa. The molar mass distribution obtained at 130 °C was very similar to that obtained at 100 °C. On the other hand, 150 and 170 °C exhibited parallel profiles that were the most different from the one obtained at 190 °C.

### 3.3. Features of Extracted Carrageenans

The carrageenan extraction yield was notably influenced by the selected temperature during the autohydrolysis treatment, which varies from 35.4 to 10.2%. The lowest extraction values were found at the lowest extraction temperature, which was 70 °C, followed by 190 °C (16.4%), 170 °C (24.3%), 80 °C (27.9%), 90 °C (28.6%), 100 °C (29.5%), 150 °C (30.2%) and 130 °C, where the highest extraction yields were identified as previously reported [31,40]. Comparable carrageenan yields have been reported from similar carrageenophyte seaweeds treated under non-alkali [32] and alkali [41] conventional procedures (<25%) and microwave hydrothermal diffusion and gravidity pre-treatment (around 30%) [35].

#### 3.3.1. Structural Features

**Figure 4:**
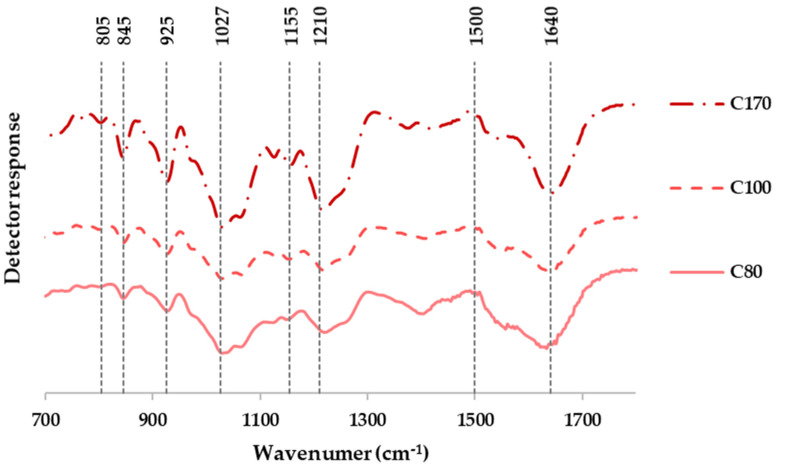
FT-IR profiles of the extracted carrageenans (C) at different temperatures using pressurized hot water extraction on the raw material, *M. stellatus*.

Figure 4 shows is representative of the Fourier-Transform Infrared spectra of extracted carrageenans from *M. stellatus* after hydrothermal treatment at different temperatures. Similar profiles were identified independently of thermal treatment. The typical kappa signal was identified at 925 cm^−1^, characteristic of the ester sulfate bonds, as well as the signal observed at 845 cm^−1^, attributed to both kappa and iota carrageenan fractions. The band found at 805 cm^−1^ has been assigned to the presence of two sulfate ester groups on the 3,6-anhydro-galactose-2-sulfate present in the iota carrageenan [40]. The iota/kappa hybridization degree (around 0.30) was calculated using the corresponding ^1^H NMR profiles, taking into account the signals at 5.2 ppm (kappa fraction) and 5.1 ppm (iota fraction). Further detail, including high-performance size exclusion chromatography, has been previously reported for these hybrid carrageenans by the authors of this work [31]; the iota/kappa hybridization degree is also in the range of those found for hybrid carrageenas isolated from similar carrageenophyte seaweeds under conventional procedures (0.45) [41].

#### 3.3.2. Rheological Features

**Figure 5:**
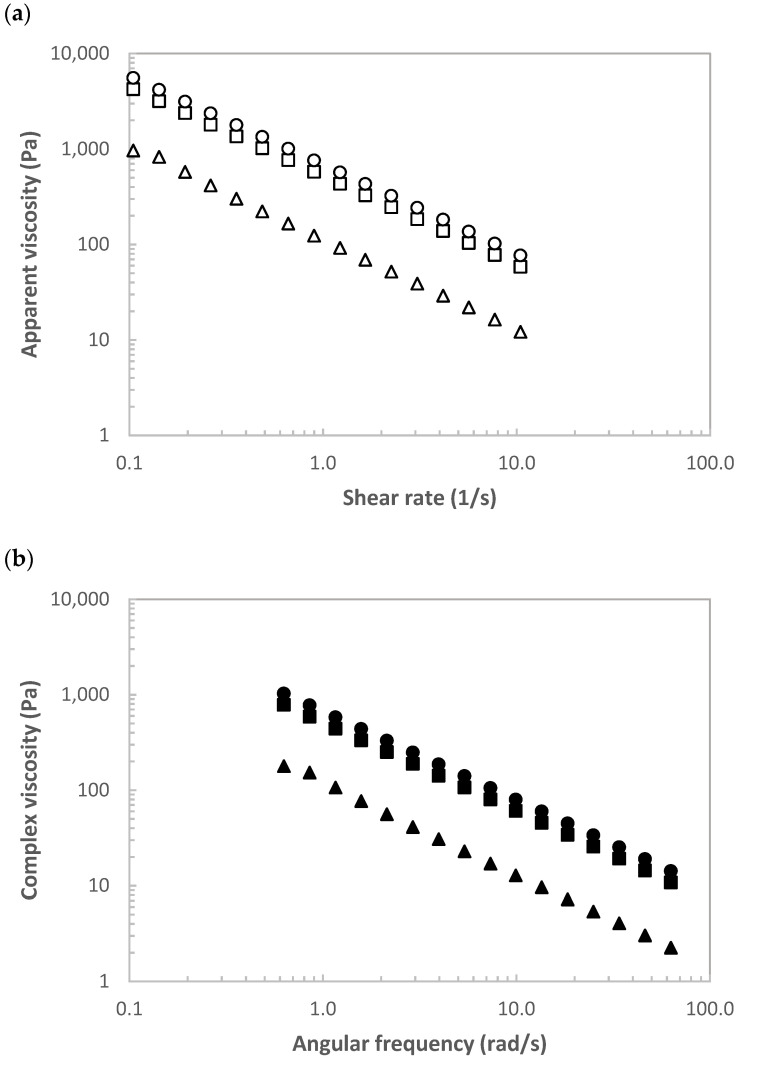
(**a**) Viscosity-shear rate curves and (**b**) complex viscosity-frequency curves at 25 °C of the representative carrageenans (C) extracted from *M. stellatus* at different temperatures (80, 100 and 170 °C) using pressurized hot water treatment formulated at 1%. Symbols: C80 (squares), C100 (circles) and C170 (triangles).

Figure 5 shows a representative of (a) viscosity-shear rate curves and (b) complex viscosity-frequency curves at 25 °C for tested hybrid carrageenan systems formulated at 1%. Note here that those prepared at 2% exhibited the same tendencies but with higher magnitudes (1.8-fold). In all cases, the apparent viscosity decreased with increasing shear rate exhibiting typical shear-thinning behavior (Figure 5a). 

At the fixed shear rate, the apparent viscosity increased for all systems prepared with carrageenans between C70 and C150 that dropped below this temperature. Similar tendencies were identified for complex viscosity profiles. This behavior is consistent with those results previously found for viscoelastic features of these hybrid carrageenans, where the gelling ability to form gels and their potential in the synthesis of nanoparticles was assessed [31]. The correspondence between experimental data from steady shear and oscillatory measurements using Cox-Merz rule was verified for tested hybrid carrageenan content throughout the similar shear-thinning behavior between dynamic and complex viscosities; however, superposition does not occur, as previously reported for other gelling polymers [42]. The magnitude of obtained values for viscoelastic moduli is comparable with those previously reported for other k/i-hybrid carrageenans under conventional treatments in the absence [32,33,34,35] and presence [41] of alkali processing during extraction.

### 3.4. Biological Activity: Antiproliferative Effect

Two cell lines, ovarian carcinoma cells (A2780) and lung carcinoma cells (A549) were used to study the cell viability for the samples: carrageenan-free liquid phases obtained by pressurized hot water and carrageenan samples after precipitation with EtOH. In the case of the extracts from *M. stellatus*, the maximum inhibitory effect was achieved for the extracts obtained at 130 °C for the A2780 cell line with the value of 65% achieving the IC_50_ at 0.31 mg/mL of extract concentration. For the A549 cell line, the result associated with the maximum inhibitory effect was observed for the extracts recovered at 100 and 130 °C with a value of 59% for both, which corresponds to 0.41 mg/mL of concentration to achieve the IC_50_. Alves et al. [43] achieved IC_50_ values for methanol extract at 0.82 mg/mL and 0.56 mg/mL using the red seaweeds *Asparagopsis armata* and *Sphaerococcus coronopifolius*, respectively, as raw material.

In relation to the results obtained for the extracted carrageenan (Table 2), the maximum inhibitory effect was exhibited at 90 °C with a value of 59%; a 0.40 mg/mL concentration was required to achieve the IC_50_ for the A2780 cell line. In the case of the lung carcinoma cell line (A549), the maximum inhibitory effect was obtained also for the carrageenan obtained at 90 °C with a result of 56%; thus, achieving the IC_50_ with a concentration of 0.50 mg/mL. In this context, similar behavior was observed in both, for the extracts and for the carrageenans, where the activity was found between 80 and 130 °C. In other works, similar results were obtained using other cell lines, e.g., human cervical carcinoma cells (HeLa) obtaining IC_50_ values of 0.55 and 0.47 mg/mL for κ- and λ-carrageenans [44]. Caco-2 (human colorectal adenocarcinoma cells) and HepG2 (human liver cancer cell lines) were selected to test commercial *k*-carrageenan by MTT assay as well. The results suggested that a higher quantity of carrageenan was required to achieve the IC_50_; for Caco-2, the IC_50_ value was 0.640 mg/mL and for HepG2, the value for IC_50_ was 0.8 mg/mL. The same behavior was observed for the other cell line, FHS 74 Int (Intestinal cell line), which required 0.88 mg/mL to achieve the IC_50_ value [45]. The results obtained in this work were consistent with the result found for Fa2n4 (human hepatocytes cell line), where the IC_50_ value was obtained at 0.42 mg/mL [45].

## 4. Conclusions

To conclude, a wide range of hybrid carrageenans from *Mastocarpus stellatus* red seaweed with attractive rheological features can be obtained, by modifying only the operational conditions of the proposed hydrothermal treatment. Operation conditions of 130 °C lead to the highest biopolymer extraction yields and the strongest viscous characteristics. A clear tendency regarding hydrothermal temperature impact was not observed in the soluble extracts of the recovered liquid phase. Another advantage of working at this temperature is the cell inhibitory effects; the maximum for A2780 is 65%, IC_50_: 0.31 mg/mL and the maximum for A549 is 59%, IC_50_: 0.41 mg/mL. The results for the antioxidant activity (as TEAC value) and total phenolic content were in harmony with the work in the bibliography. In this context, the results of this work suggest that the ecofriendly extraction technology could be a smart strategy for producing carrageenan polymers that are of potential interest to industries such as the pharmaceutical, food or cosmetic. Furthermore, future trends to broaden the behavior in biological assays as antiviral activity could be of interest.

## Figures and Tables

**Table 1:** Chemical and bioactive features of the carrageenan-free liquid phase extracted using hot water extraction at different temperatures.

T (°C)	pH (-)	Dry Content (%)	TEAC Value (%)	Phenolic Content (%)	Sulfate Content (%)	Protein Content (%)	Oligosaccharide Content (%)
70	7.35 ± 0.04	0.32 ± 0.02 ^f^	3.21 ± 0.15 ^b^	0.25 ± 0.01 ^a^	23.92 ± 0.37 ^g^	0.95 ± 0.13 ^a^	27.05 ± 0.17
80	7.49 ± 0.02	0.36 ± 0.01 ^f^	3.33 ± 0.12 ^b^	0.24 ± 0.01 ^a^	44.29 ± 0.26 ^c^	0.98 ± 0.12 ^a^	34.87 ± 0.12
90	7.50 ± 0.01	0.34 ± 0.01 ^f^	3.76 ± 0.13 ^a^	0.24 ± 0.01 ^a^	45.67 ± 0.35 ^b^	1.15 ± 0.12 ^a^	43.00 ± 0.15
100	6.85 ± 0.04	0.67 ± 0.01 ^e^	2.37 ± 0.36 ^c^	0.16 ± 0.01 ^b^	39.09 ± 0.35 ^e^	1.27 ± 0.08 ^a^	47.30 ± 0.07
130	7.04 ± 0.01	1.76 ± 0.05 ^d^	1.26 ± 0.10 ^e^	0.04 ± 0.01 ^d^	36.48 ± 0.53 ^f^	0.79 ± 0.10 ^b^	57.88 ± 0.18
150	6.98 ± 0.02	2.32 ± 0.01 ^c^	1.87 ± 0.04 ^d^	0.05 ± 0.01 ^d^	41.31 ± 0.21 ^d^	0.71 ± 0.04 ^b^	60.41 ± 0.09
170	6.73 ± 0.01	2.64 ± 0.03 ^a^	1.53 ± 0.13 ^e^	0.08 ± 0.01 ^c,d^	47.80 ± 0.82 ^a^	0.75 ± 0.03 ^b^	61.59 ± 0.15
190	6.17 ± 0.01	2.55 ± 0.01 ^b^	3.64 ± 0.17 ^a^	0.13 ± 0.01 ^c^	11.44 ± 0.59 ^h^	0.84 ± 0.03 ^b^	44.33 ± 0.23

Data are presented as mean ± standard deviation. Data values in a column with different superscript letters are significantly different at the *p* ≤ 0.05 level.

**Table 2:** Antiproliferative behavior of recovered soluble extracts free of carrageenan obtained by pressurized hot water extraction (E) and the corresponding hybrid carrageenans recovery by precipitation with EtOH (C) for two human tumor cell lines: ovarian carcinoma (A2780) and lung carcinoma (A549). Note: the number after the letters E and C represent the extraction temperature.

	A2780		A549	
Sample	E_max_ (% Inhibition)	IC_50_ (mg/mL)	E_max_ (% Inhibition)	IC_50_ (mg/mL)
E70	39	-	26	-
E80	55	0.40 ± 0.01	51	0.50 ± 0.01
E90	60	0.34 ± 0.01	58	0.45 ± 0.00
E100	63	0.33 ± 0.00	59	0.41 ± 0.01
E130	65	0.31 ± 0.01	59	0.41 ± 0.01
E150	33	-	28	-
E170	19	-	12	-
E190	13	-	8	-
C70	48	-	35	-
C80	52	0.43 ± 0.01	50	0.53 ± 0.01
C90	59	0.40 ± 0.00	56	0.50 ± 0.00
C100	58	0.41 ± 0.01	54	0.50 ± 0.01
C130	50	0.44 ± 0.01	48	-
C150	23	-	16	-
C170	12	-	9	-
C190	4	-	2	-

## Data Availability

The data presented in this study are available on request from the corresponding author.
